# LncRNA MNX1-AS1 drives the progression of non-small cell lung cancer and serves as a ceRNA to target COMMD8 by sponging miR-218-5p

**DOI:** 10.1007/s10735-026-10891-3

**Published:** 2026-07-29

**Authors:** Gao-Feng Liu, Li Zhou, Yu-Bo Zhao, Jia-Hong Tang, Qing-Yuan Li, Xiao-Yong Ding, Xiao-Zhen Zhang, Su-Juan Cui, Yong Zhang, Zhen-Wei Sun

**Affiliations:** 1 Department of Cardiothoracic Surgery, The 988th Hospital of PLA Joint Logistics Support, Zhengzhou, Henan Province 450042 China; 2 Department of Oncology, The 988th Hospital of PLA Joint Logistics Support, Zhengzhou, Henan Province 450042 China; 3 Department of Laboratory and Blood Transfusion, The 988th Hospital of PLA Joint Logistics Support, Zhengzhou, Henan Province 450042 China

**Keywords:** MNX1-AS1, miR-218-5p, COMMD8, Non-small cell lung cancer, Proliferation, Migration

## Abstract

The role and mechanistic action of MNX1-AS1 in non-small cell lung cancer (NSCLC) remain unclear. This study aimed to investigate the function of MNX1-AS1 in the progression of NSCLC. A total of 40 paired tumor and adjacent lung tissue samples from patients with NSCLC were collected to investigate the expression of MNX1-AS1. H1299 and HCC827 cells were transfected with sh-MNX1-AS1, shRNA-NC, miR-218-5p mimic, mimic-NC, pcDNA3.1-COMMD8, or empty vector (pcDNA3.1). The expression of MNX1-AS1, miR-218-5p, and COMMD8 was detected using quantitative real-time PCR (qRT-PCR). Cell proliferation was evaluated using CCK-8 assays, while cell migration and invasion were evaluated using Transwell assays. Dual-luciferase reporter and RNA pull-down assays were performed to validate the targeting interactions between MNX1-AS1 and miR-218-5p, as well as between COMMD8 and miR-218-5p. In vivo, a nude mouse model was established: tumor volume and weight were measured after four weeks. Gene expression in tumor tissue was quantified using qRT-PCR, and Ki-67 expression was evaluated using immunohistochemistry. MNX1-AS1 expression was significantly upregulated in NSCLC tissues (*P* < 0.05) and was correlated with poor prognosis in NSCLC patients. MNX1-AS1 knockdown markedly suppressed the viability, migration, and invasion of H1299 and HCC827 cells (*P* < 0.05). Mechanistically, MNX1-AS1 acted as a molecular sponge for miR-218-5p, thereby relieving miR-218-5p-mediated repression of its direct target COMMD8 (*P* < 0.05). Functional rescue experiments revealed that miR-218-5p silencing or COMMD8 overexpression partially reversed the inhibitory effects of MNX1-AS1 knockdown on the cell viability, migration, and invasion of H1299 and HCC827 cells. In vivo, sh-MNX1-AS1 significantly reduced tumor volume and weight (*P* < 0.05). Moreover, the expression of MNX1-AS1, COMMD8, and Ki-67 were downregulated, while the expression of miR-218-5p was upregulated in sh-MNX1-AS1 tumor tissues (*P* < 0.05). MNX1-AS1 promotes the progression of NSCLC by modulating the miR-218-5p/COMMD8 axis.

## Introduction

Lung cancer is the most lethal malignancy worldwide, accounting for 18% of all cancer-related deaths (Sung et al. 2021). Non-small cell lung cancer (NSCLC), the predominant subtype of lung cancer, accounts for approximately 80% of all cases, with a five-year survival rate of only 10%-24% (Kumar and Sarkar 2022; Sung et al. 2021). NSCLC is primarily classified into lung adenocarcinoma (LUAD), lung squamous cell carcinoma (LSCC), and large cell carcinoma, with LUAD and LSCC being the most prevalent subtypes (Sung et al. 2021). Consequently, identifying effective therapeutic targets for the treatment of NSCLC is crucial.

Long non-coding RNAs (lncRNAs) are a class of non-protein-coding RNA molecules with a length of more than 200 nucleotides. Accumulating studies have demonstrated lncRNAs can precisely regulate gene expression through multiple mechanisms, including the recruiting transcription factors, participating in stress granules assembly, and acting as molecular sponges (Wang et al. 2017, 2021; Xia et al. 2022), thereby driving the initiation and progression of malignant tumors (Wang et al. 2017, 2023a; Zhang et al. 2023). As a vital oncogenic lncRNA, MNX1-AS1 is aberrantly upregulated in a variety of human cancers and facilitates tumor progression by activating proliferation-related signaling pathways, promoting cell migratory and invasive, and suppressing apoptosis. Clinically, elevated MNX1-AS1 expression is correlated with poor prognosis (Cui et al. 2021; Li et al. 2022; Liu et al. 2019c; Shen and Zhou 2020; Shuai et al. 2020). Specifically, MNX1-AS1 exerts oncogenic functions in lung cancer. Knockdown of MNX1-AS1 significantly suppresses the proliferation and metastasis of lung cancer cells while inducing cell apoptosis (Liu et al. 2019a, 2022, 2019b).

Micro-RNAs (miRNAs) are a class of 19–24 nucleotides non-coding RNAs that negatively regulate gene expression by promoting mRNA degradation or suppressing its translation. Previous research has confirmed that miRNAs are extensively involved in critical biological processes, including cell proliferation, apoptosis, and migration (Hao et al. 2023; Li et al. 2019). In human cancers, miRNAs exert dual biological functions, acting as either tumor suppressors or oncogenes depending on their expression levels and downstream target genes (Kittelmann and McGregor 2019; Mohamed et al. 2020). Accumulating evidence has indicated that miR-218-5p plays a tumor-suppressive role in multiple human malignancies, including renal cell carcinoma, hepatocellular carcinoma, as well as bladder, colorectal, breast and gastric cancers(Chang et al. 2023; Chu et al. 2023; Shiomi et al. 2019; Wang et al. 2020b; Xie et al. 2020; Zhu et al. 2020). Moreover, miR-218-5p has been reported to possess potent tumor-suppressive properties in NSCLC (Wang et al., 2023b; Zhu et al. 2016). However, whether MNX1-AS1 promotes NSCLC progression by sponging miR-218-5p remains unclear, and the underlying functional pathways and regulatory targets of MNX1-AS1 require further investigation.

COMMD8, a member of the COMMD protein family, is aberrantly overexpressed in NSCLC tissues and cells. COMMD8 depletion markedly inhibits the proliferation and colony formation of NSCLC cells, suggesting that COMMD8 acts as a crucial oncogenic driver in NSCLC progression (Wang et al. 2020a). Another study has demonstrated that COMMD8 overexpression partially reverses the inhibitory effects of LINC00657 knockdown on the proliferation and migration of NSCLC cells (Zhang et al. 2020). However, the upstream regulatory mechanisms governing COMMD8 expression have yet to be elucidated. In particular, it remains unclear whether COMMD8 is regulated by miR-218-5p and participates in the oncogenic effects mediated by the MNX1-AS1/miR-218-5p axis in NSCLC.

Accordingly, the present study focused on three objectives: (1) to investigate the expression characteristics and clinical significance of MNX1-AS1 in NSCLC; (2) to explore whether MNX1-AS1 regulates COMMD8 expression by sponging miR-218-5p; and (3) to elucidate the biological function of MNX1-AS1/miR-218-5p/COMMD8 axis in NSCLC malignant progression, providing a new theoretical basis for molecular targeted therapy for NSCLC.

## Materials and methods

### Tissue samples

A total of 40 paired NSCLC and adjacent normal lung tissues were obtained from patients who underwent surgical resection at 988th Hospital of PLA Joint Logistics Support Force from June 2019 to June 2022. Complete clinical dataset was obtained for all patients, and none had received chemotherapy, radiotherapy, or immunotherapy before surgery. All patients were followed up regularly until June 2024. Overall survival time was calculated from the date of surgery to death. Clinicopathological and follow-up data were statistically analyzed. The study was approved by the Medical Ethics Committee of the 988th Hospital of PLA Joint Logistics Support Force (No.988YY20230002LLSP). Written informed consent was acquired from all patients.

### Cell lines and culture

Two NSCLC cell lines, H1299 (RRID:CVCL_0060) and HCC827 (RRID:CVCL_2063), were purchased from the Shanghai Cell Bank of the Chinese Academy of Sciences. All cells were authenticated by short tandem repeat (STR) profiling, with results consistent with the standard database, and tested negative for mycoplasma contamination. Certificates for STR authentication and mycoplasma testing are provided in the supplementary file. Cells were cultured in RPMI-1640 medium (Thermo Fisher Scientific, Waltham, MA, USA), supplemented with 10% fetal bovine serum (FBS, Gibco, USA) and maintained in a humidified incubator with 5% CO_2_ at 37 ℃.

### Transfection

Short hairpin RNAs (shRNAs) targeting MNX1-AS1, pcDNA3.1-COMMD8 overexpression vector (OE-COMMD8), miR-218-5p mimic and miR-218-5p inhibitor, as well as their corresponding negative controls (shRNA-NC, pcDNA3.1 empty vector, mimic-NC and inhibitor-NC), were purchased from GenePharma Co., Ltd (Shanghai, China). Transfection of sh-MNX1-AS1 (or shRNA-NC), miR-218-5p mimic (or mimic-NC), and pcDNA3.1-COMMD8 (or empty pcDNA3.1 vector) was carried out using Lipofectamine 3000 Reagent (Thermo Fisher, USA) following the manufacturer’s instructions. The sequences of sh-MNX1-AS1 and shRNA-NC are listed in Supplementary Table.

### Quantitative real-time PCR (qRT-PCR) assay

Total RNA was extracted using TRIzol reagent (Thermo Fisher, USA) and reverse-transcribed into cDNA with the PrimeScript™ RT reagent Kit (Takara, Japan). qRT-PCR was carried out with the SYBR Premix Ex Taq Ⅱ Kit on a 7900HT Fast Real-Time PCR System (Applied Biosystems, USA) to detect the expression of *MNX1-AS1, miR-218-5p* and *COMMD8*. *U6* and *GAPDH* were used as internal reference genes. Relative expression levels were calculated using the 2^−∆∆Ct^ method.

### CCK-8 assays

Cell proliferation was evaluated using Cell Counting Kit-8 (CCK-8, MedChemExpress, Shanghai, China) following the manufacturer’s instructions. NSCLC cells of each group were seeded into 96-well plates at a density of 5 × 10^3^ cells per well, with three replicate wells. After incubation for 24, 48, and 72 h, 10 μL CCK-8 solution was added into each well, and the plates were incubated for another 4 h. The optical density (OD) at 450 nm was then measured using a microplate reader (Thermo Fisher, USA).

#### Transwell assays

Cell migration was examined using Matrigel-free Transwell chambers (Corning, USA). For invasion assays, chambers were pre-coated with Matrigel. Briefly, 1 × 10^5^ cells in serum-free RPMI-1640 medium were seeded into the upper chamber. The lower chambers were filled with complete RPMI-1640 medium supplemented with 20% FBS. After 24 h incubation, migrated and invasive cells were fixed with methanol. Cells adhering to the upper surface of the membrane were wiped off using cotton swabs. Cells were stained with 0.1% crystal violet (Solarbio Co., Ltd., Beijing, China). Finally, five random microscopic fields per group were photographed, and the cell numbers and quantified.

#### Dual-luciferase reporter gene assays

Potential binding between MNX1-AS1 and miR-218-5p was predicted using LncBase V3.0 (https://diana.e-ce.uth.gr/lncbasev3), while COMMD8 was predicted as a target of miR-218-5p by StarBase (https://rnasysu.com/encori/). Wild-type (WT) and mutant (MUT) fragments of MNX1-AS1 and COMMD8 were synthesized, their sequences are listed in the Supplementary Table. WT sequences of MNX1-AS1 and the COMMD8 3' UTR containing predicted miR-218-5p binding sites were cloned into the pmiR-GLO vector (Invitrogen, USA). Site-directed mutagenesis was performed to construct corresponding MUT fragments, in which the key complementary sequences were mutated to abolish the binding capability of miR-218-5p. The recombinant pmiR-GLO vectors were co-transfected with the miR-218-5p mimic or miR-NC into HEK-293 T cells using Lipofectamine 3000. At 48 h post-transfection, relative luciferase activity was measured with the Dual-Luciferase Reporter (DLR™) Assay System (Promega, USA) according to the manufacturer's instructions.

#### RNA pull-down assays

Biotinylated miR-218-5p wild-type (bio-miR-218-5p-wt), mutant (bio-miR-218-5p-mut), and negative control (bio-NC, a sequence unrelated to known mammalian miRNAs) probes were synthesized by GenePharma (Shanghai, China) and transfected into NSCLC cells separately. Cells were collected 48 h post-transfection and washed twice with ice-cold PBS. For lysis, 1 × 10^7^ cells were resuspended in 500 µL of ice-cold lysis buffer (20 mM Tris–HCl pH 7.5, 150 mM NaCl, 0.5 mM EDTA, 0.5% NP-40, 1 U/μL RNase inhibitor, and 1 mM DTT) and incubated on ice for 30 min. Lysates were centrifuged at 13,000 × g for 10 min at 4 °C, and the supernatant was retained for further analysis. M-280 Streptavidin magnetic beads (Invitrogen, USA) were magnetically separated and washed twice with 500 µL of ice-cold binding/washing buffer (10 mM Tris–HCl pH 7.5, 1 mM EDTA, 2 M NaCl). A total of 40 pmol of biotin-labeled probes were incubated with beads at room temperature under rotation for 20 min to facilitate conjugation. Following magnetic separation, beads were rinsed with binding buffer and resuspended in an equal volume of the buffer. Beads coupled with probes were mixed with cell lysate at a 1:1 ratio and rotated overnight at 4 °C. The beads were then magnetically separated and washed 3–5 times with 500 µL ice-cold washing buffer (50 mM Tris–HCl pH 7.5, 300 mM NaCl, 0.1% NP-40), followed by a final rinse in 1 × PBS. Bound products were eluted by 80 µL of elution buffer (10 mM Tris–HCl pH 7.5, 1 mM EDTA, 2% SDS) and heating at 95 °C for 5–10 min. After magnetic separation, the eluate was harvested. Total RNA was extracted from the eluate with TRIzol reagent, and *MNX1-AS1* expression was measured by qRT-PCR.

### Western blotting

Total protein was extracted using RIPA lysis buffer supplemented with 0.1 mM phenylmethylsulfonyl fluoride (PMSF, Beyotime, Jiangsu, China). Protein samples (40 μg) were separated by 10% sodium dodecyl sulfate–polyacrylamide gel electrophoresis (SDS-PAGE) and electrotransferred onto polyvinylidene difluoride membranes (PVDF, Millipore, USA). After blocking with 5% non-fat milk (Beyotime, Jiangsu, China), the membranes were incubated with primary antibodies (Proteintech, Wuhan, China) against COMMD8 (1:1000) and β-actin (1:5000) overnight at 4 ℃. Following several washes, membranes were incubated with secondary antibodies (Thermo Fisher, USA) for 1 h at room temperature. Protein bands were detected using a chemiluminescence detection reagent (Cell Signaling Technology, USA). Relative expression of COMMD8 was quantified using ImageJ software (National Institutes of Health, USA).

### Xenograft models

All animal procedures were approved by the Animal Research Ethics Committee of the 988th Hospital of PLA Joint Logistic Support Force (Zhengzhou, China). Ten 5–6-weeks-old BALB/c nude mice were obtained from the Laboratory Animal Center of Henan Province (Zhengzhou, China). The sample size calculation is provided in Supplementary Figure. After one week of acclimatization, mice were randomly assigned to two groups (*n* = 5 per group) using a random number table. Under blinded conditions, 3 × 10⁶ H1299 cells stably transfected with sh-MNX1-AS1 or shRNA-NC were subcutaneously injected into the right axillary fossa of each mouse. Tumor volumes were measured weekly and calculated using the formula: volume (V) = width^2^ × length × 0.5. Four weeks after inoculation, all mice were euthanized, and tumors were harvested and weighed.

### Immunohistochemistry

Portions of tumor tissues were reserved for qRT-PCR detection. The remaining tumor tissues were fixed in 4% formalin for paraffin embedding and sectioning at 4 μm. After blocking endogenous peroxidase activity, sections were incubated with anti-Ki-67 antibody (Abcam, UK) overnight at 4 ℃. Sections were then incubated with HRP-conjugated secondary antibodies (Thermo Fisher Scientific, USA) at 37 ℃ for 1 h, visualized with 3,3-diaminobenzidine solution (DAB, Solarbio, China) for 3 min, followed by counterstaining with hematoxylin.

### Statistical analysis

All data were analyzed using SPSS Statistics 26.0 (IBM). Data are presented as mean ± standard deviation (SD). One‑way analysis of variance (ANOVA) with Bonferroni post‑hoc tests was used for multiple group comparisons. Categorical data were analyzed by χ^2^ test. Kaplan–Meier survival curves were constructed and the log-rank test was used for intergroup comparison. Bonferroni correction was applied to adjust P values in multiple pairwise comparisons during univariate Cox regression. Univariate Cox proportional hazards regression was used to calculate hazard ratios (HRs) and 95% confidence intervals (CIs). *P* < 0.05 was considered statistically significant.

## Results

### MNX1-AS1 was upregulated in NSCLC tissues and correlated with a poor prognosis

**Fig. 1:**
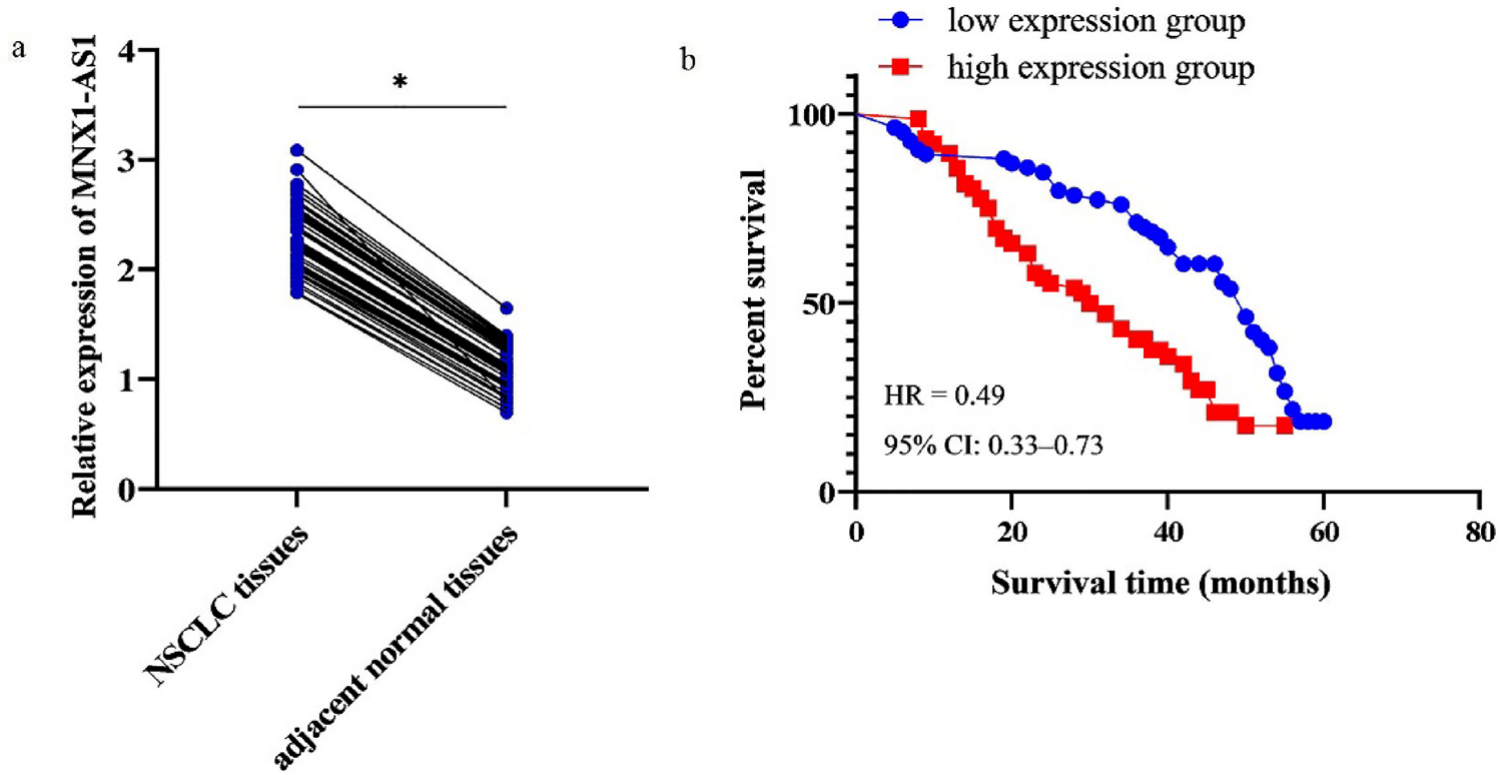
MNX1-AS1 expression is upregulated in NSCLC tissues and correlated with poor prognosis. **a**: MNX1-AS1 expression in 40 pairs of NSCLC tumor tissues and paired adjacent normal lung tissues was detected by qRT-PCR. n = 40, error bars indicate standard deviation (SD). ^*^P < 0.05 *vs* adjacent normal tissues. **b**: Patients were divided into high-expression (n = 20) and low-expression (n = 20) groups according to the mean expression of MNX1-AS1 in NSCLC tissues. Overall survival was analyzed using Kaplan–Meier survival curves, and the Log-rank test was used for statistical comparison

The expression of MNX1-AS1 in NSCLC and paired adjacent normal tissues was detected via qRT-PCR (Fig. 1a). MNX1-AS1 was significantly upregulated in NSCLC tissues (*P* < 0.05). Using the mean expression of MNX1-AS1 in NSCLC tissues as the cut-off, patients were divided into high- and low-expression groups for the analysis of clinicopathological features and prognosis. Correlation analysis revealed that high MNX1-AS1 expression was associated with poor tumor differentiation, advanced TNM stage and increased lymph node metastasis (Table 1, *P* < 0.05). Kaplan–Meier curves revealed that patients with high MNX1-AS1 expression had worse overall survival (Log-rank χ^2^ = 16.08, *P* < 0.05, Fig. 1b).

**Table 1:** Association of MNX1-AS1 with clinicopathologic factors in patients with NSCLC

Variables	No. of cases	MNX1-AS1 expression		*P* value^a^
		Low	High	
Sex				*P* = 0.816
Male	29	17	12	
Female	11	6	5	
Differentiation				*P* = 0.035
Low	15	9	6	
Medium	14	11	3	
High	11	3	8	
TNM stage				*P* = 0.025
Ⅰ/Ⅱ	20	15	5	
Ⅲ/Ⅳ	20	8	12	
Lymph node metastasis				*P* = 0.012
Yes	19	7	12	
No	21	16	5	

### MNX1-AS1 knockdown inhibited the viability, migration and invasion of NSCLC cells

**Fig. 2:**
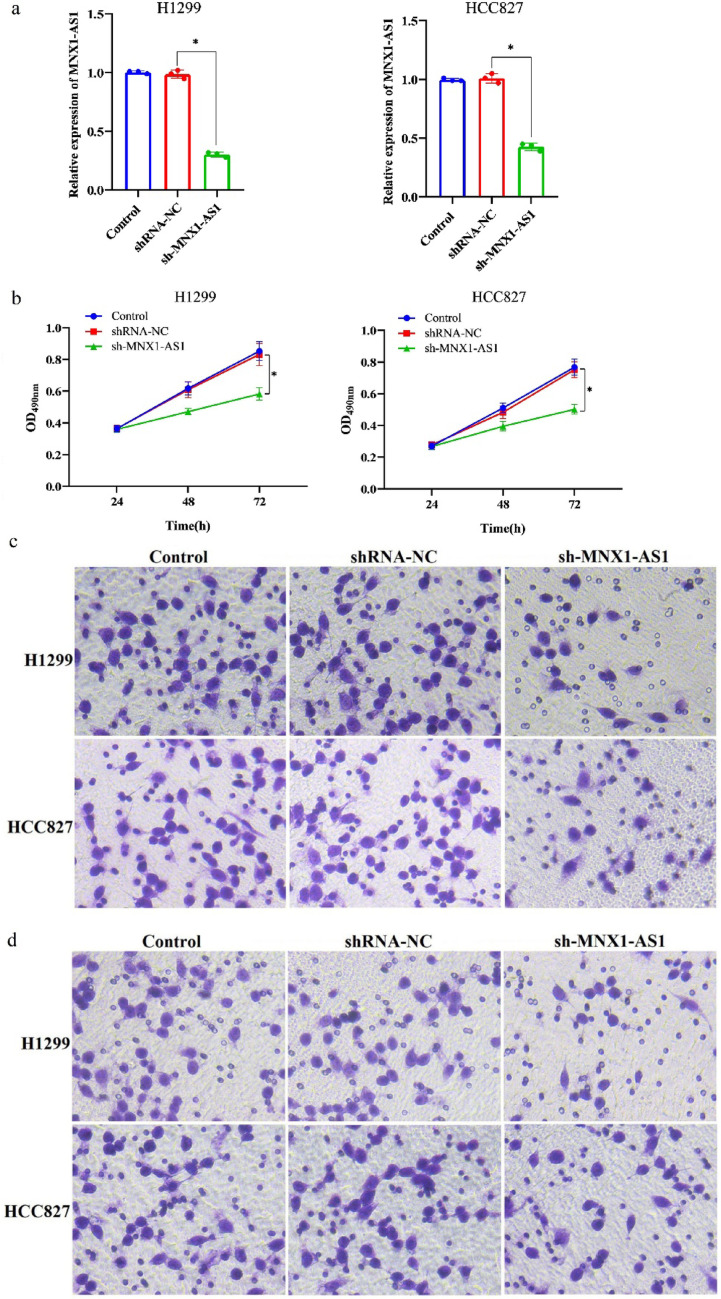
MNX1-AS1 knockdown suppresses the viability, migration and invasion of NSCLC cells. **a**: qRT-PCR was used to detect MNX1-AS1 expression in H1299 and HCC827 cells 48 h after transfection with sh-MNX1-AS1 or shRNA-NC. **b**: CCK-8 assays were performed to assess cell viability at 24, 48, and 72 h post-transfection. **c**-**d**: Transwell assays were used to assess cell migration and invasion at 48 h post-transfection. n = 3, error bars indicate SD. ^*^P < 0.05 *vs* shRNA-NC

Loss-of-function assays were conducted to explore the role of MNX1-AS1 in the proliferation, migration and invasion of NSCLC cells. Transfection of sh-MNX1-AS1 efficiently downregulated MNX1-AS1 expression in H1299 and HCC827 cells (Fig. 2a). Knockdown of MNX1-AS1 significantly inhibited cell viability, migration and invasion in both cell lines (*P* < 0.05, Fig. 2b–d). These results demonstrated that MNX1-AS1 promoted the proliferative and metastatic potential of NSCLC cells.

### MNX1-AS1 sponged miR-218-5p in NSCLC cells

**Fig. 3:**
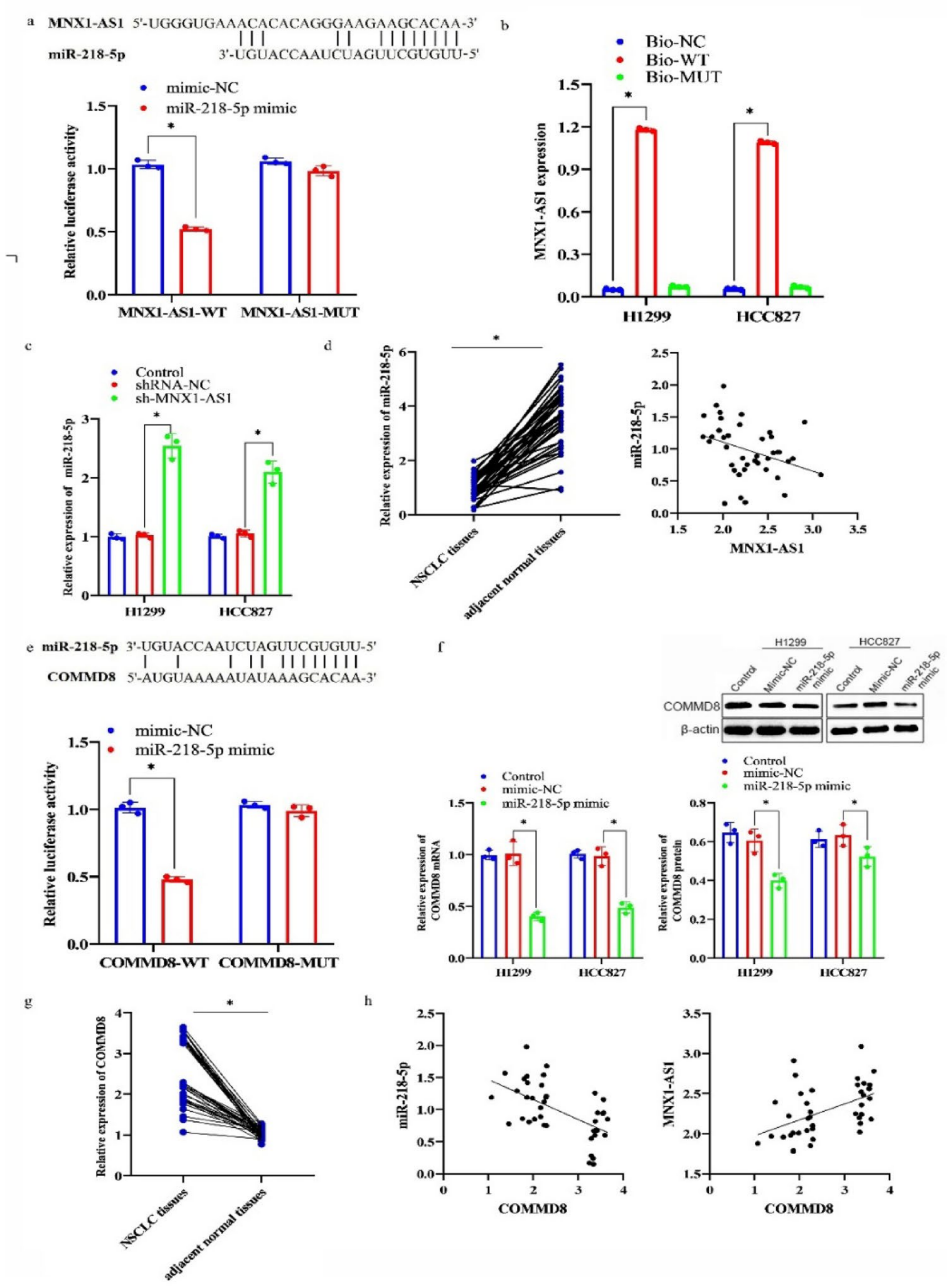
MNX1-AS1/miR-218-5p/COMMD8 regulatory axis in NSCLC cells **a**: Potential binding between MNX1-AS1 and miR-218-5p was predicted using lncBase V3.0 and further verified by luciferase reporter assays. Luciferase activity was measured at 48 h post-transfection. n = 3, error bars indicate SD. ^*^P < 0.05 *vs* mimic-NC. **b**: RNA pull-down assays confirmed the interaction between MNX1-AS1 and miR-218-5p in NSCLC cells. qRT-PCR was performed to detect MNX1-AS1 enrichment at 48 h post-transfection. n = 3, error bars indicate SD. ^*^P < 0.05 *vs* Bio-NC. **c**: Knockdown of MNX1-AS1 increased miR-218-5p expression in H1299 and HCC827 cells. n = 3, error bars indicate SD. ^*^P < 0.05 *vs* shRNA-NC. **d**: miR-218-5p is significantly downregulated and shows a negative correlation with MNX1-AS1 in NSCLC tissues. Left: qRT-PCR analysis of miR-218-5p expression in 40 pairs of NSCLC tumor tissues and paired adjacent normal lung tissues. ^*^P < 0.05 *vs* adjacent normal tissues. Right: Pearson correlation analysis of MNX1-AS1 and miR-218-5p expression in NSCLC tissues. n = 40, error bars indicate SD. **e**: The binding relationship between miR-218-5p and COMMD8 was predicted using StarBase and validated by luciferase reporter assays. Luciferase activity was measured 48 h after transfection. n = 3, error bars indicate SD. ^*^P < 0.05 *vs* mimic-NC. **f**: miR-218-5p overexpression reduced COMMD8 expression in H1299 and HCC827 cells. The mRNA and protein levels of COMMD8 were detected by qRT-PCR and Western blot, respectively, at 48 h post-transfection. n = 3, error bars indicate SD. ^*^P < 0.05 *vs* mimic-NC. **g**: COMMD8 was markedly upregulated in NSCLC tissues. qRT-PCR was used to detect COMMD8 expression in 40 pairs of NSCLC tumor tissues and paired adjacent normal lung tissues. n = 40, error bars indicate SD. ^*^P < 0.05 *vs* adjacent normal tissues. **h**: Pearson correlation analysis showed that miR-218-5p was negatively correlated with COMMD8, while MNX1-AS1 was positively correlated with COMMD8 in NSCLC tissues

LncRNAs can function as competing endogenous RNAs (ceRNAs) to sequester miRNAs (Salmena et al. 2011). To investigate the molecular mechanism of MNX1-AS1 in NSCLC, bioinformatics analysis was performed to screen potential miRNA targets of MNX1-AS1 and clarify the underlying regulatory pathways. Potential binding sites between MNX1-AS1 and miR-218-5p were predicted by LncBase V3.0 (Fig. 3a). Luciferase activity of the MNX1-AS1-WT vector was markedly decreased after transfection with the miR-218-5p mimic, while the change was not detected in the MNX1-AS1-MUT group in HEK-293 T cells (*P* < 0.05, Fig. 3a). RNA pull-down assays further confirmed the interaction between MNX1-AS1 and miR-218-5p. MNX1-AS1 was enriched by bio-miR-218-5p-wt, while bio-miR-218-5p-mut failed to bind MNX1-AS1 in H1299 and HCC827 cells (Fig. 3b). Knockdown of MNX1-AS1 enhanced miR-218-5p expression in the two cell lines (*P* < 0.05, Fig. 3c). Moreover, miR-218-5p was significantly downregulated in NSCLC tissues and negatively correlated with MNX1-AS1 expression (*P* < 0.05, Fig. 3d). All data indicate that MNX1-AS1 targets and sponges miR-218-5p in NSCLC cells.

### COMMD8 was identified as a target gene of miR-218-5p

Bioinformatics analysis predicted specific binding sites between miR-218-5p and COMMD8 (Fig. 3e). Luciferase reporter assays demonstrated that the miR-218-5p mimic significantly suppressed the luciferase activity of COMMD8-WT, whereas no significant change was detected in the COMMD8-MUT group (*P* < 0.05, Fig. 3e). miR-218-5p overexpression downregulated COMMD8 protein levels in H1299 and HCC827 cells (*P* < 0.05, Fig. 3f). Moreover, COMMD8 expression was upregulated in NSCLC tissues, with a negative correlation with miR-218-5p, and a positive correlation with MNX1-AS1 (*P* < 0.05, Fig. 3g–h). These results demonstrate that COMMD8 is a direct downstream target of miR-218-5p and negatively regulated by miR-218-5p.

### MNX1-AS1 exerted an oncogenic role in NSCLC via the miR-218-5p/COMMD8 axis

**Fig. 4:**
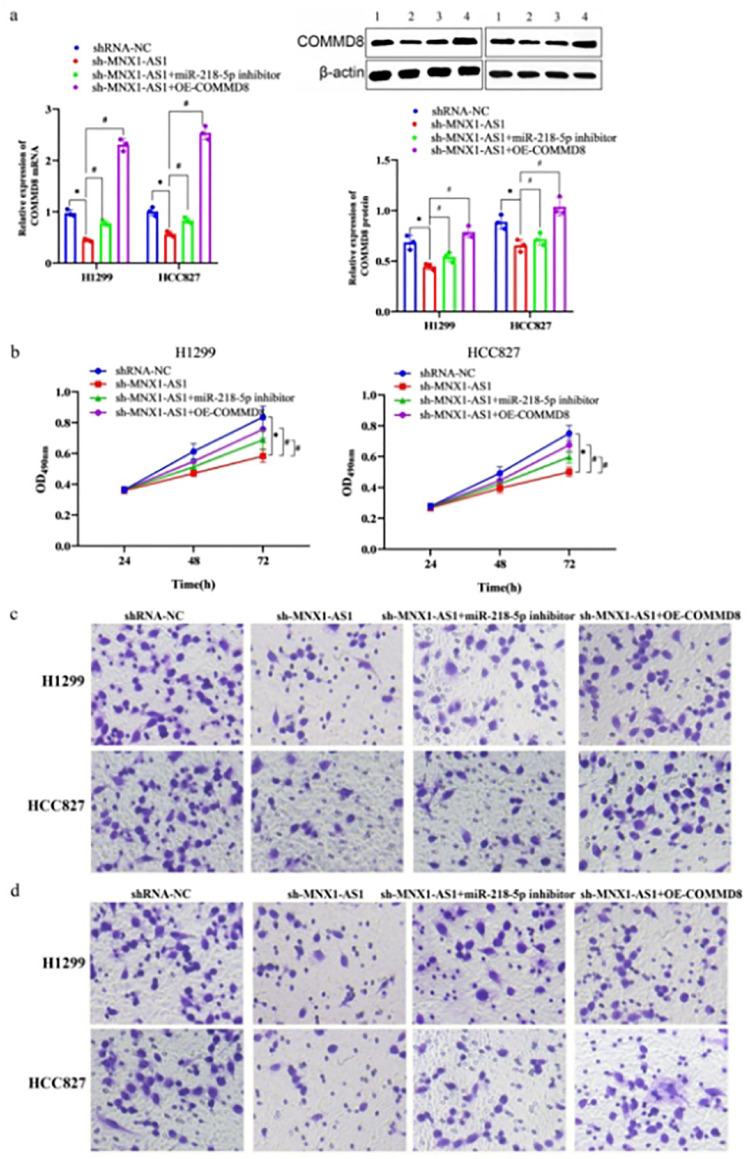
MNX1-AS1 promotes NSCLC tumorigenesis via the miR-218-5p/COMMD8 axis. **a**: qRT-PCR and Western blot were used to detect COMMD8 expression in H1299 and HCC827 cells at 48 h post-transfection. Four experimental groups were set: shRNA-NC (1), sh-MNX1-AS1 (2), sh-MNX1-AS1 + miR-218-5p inhibitor (3), and sh-MNX1-AS1 + OE-COMMD8 (4). **b**: CCK-8 assays were performed to assess cell viability in the four groups at 24, 48, and 72 h post-transfection. c-d: Transwell assays were applied to evaluate cell migration **c** and invasion **d** in the four groups at 48 h post-transfection. All data were presented as bar graphs. n = 3, error bars indicate SD. ^*^P < 0.05 *vs* shRNA-NC, ^#^*P* < 0.05 *vs* sh-MNX1-AS1

The regulatory effects of the MNX1-AS1/miR-218-5p/COMMD8 axis on cell viability, migration, and invasion were explored in H1299 and HCC827 cells. qRT-PCR and Western blot revealed that COMMD8 expression was decreased after sh-MNX1-AS1 transfection, and this phenotype was partially restored by miR-218-5p inhibitor or COMMD8 overexpression (*P* < 0.05, Fig. 4a). The inhibitory effects on cell viability, migration, and invasion induce by MNX1-AS1 knockdown were also partially reversed by miR-218-5p silencing or COMMD8 overexpression (*P* < 0.05, Fig. 4b–d). Taken together, MNX1-AS1 exerts oncogenic effects in NSCLC by modulating the miR-218-5p/COMMD8 axis.

### MNX1-AS1 knockdown impaired tumorigenesis in vivo

**Fig. 5:**
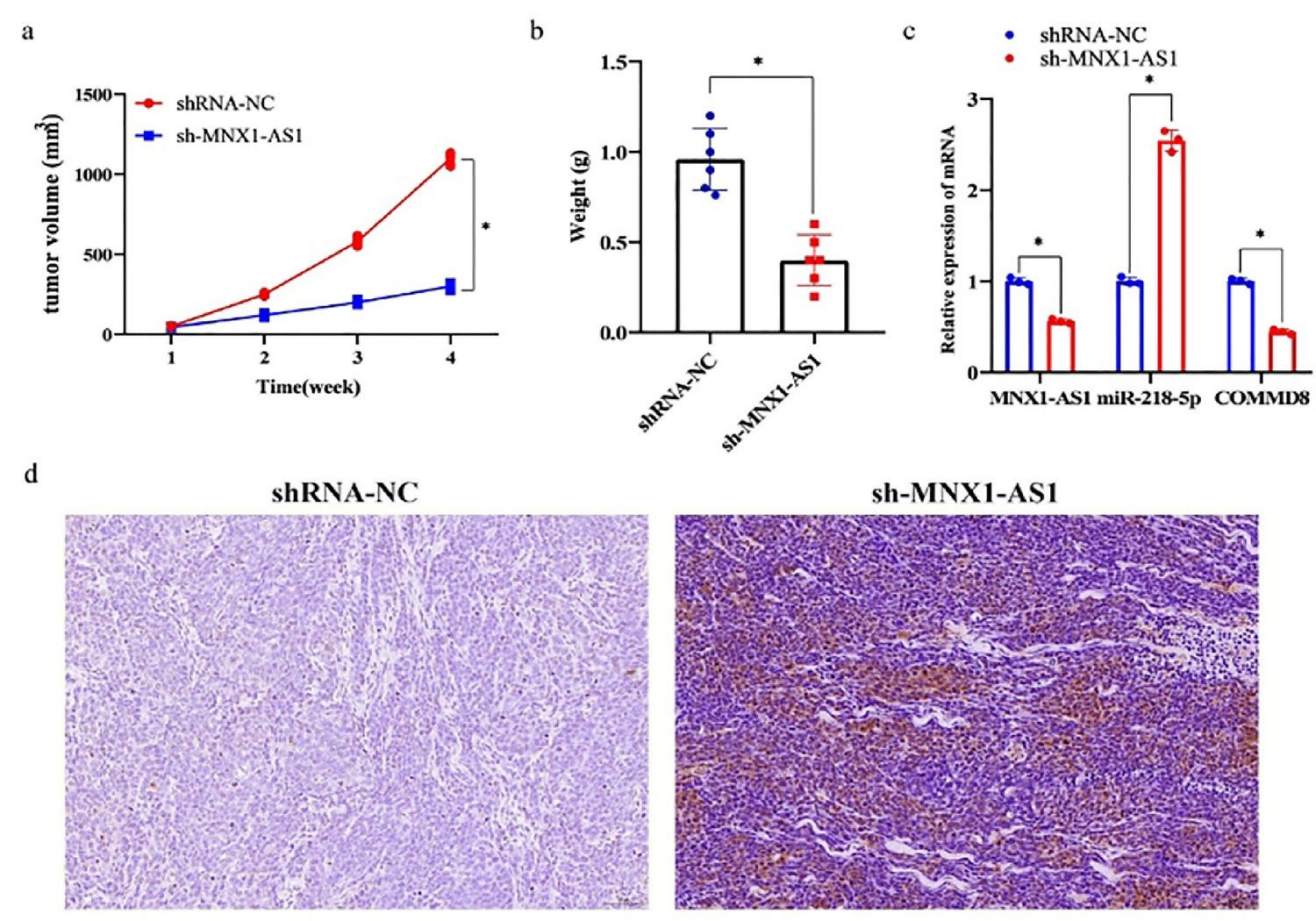
MNX1-AS1 accelerates NSCLC progression in vivo*.* **a**-**b**: Knockdown of MNX1-AS1 suppressed tumor growth in BALB/c nude mice subcutaneously injected with H1299 cells stably expressing sh-MNX1-AS1 or shRNA-NC. Tumor volume was measured every 7 days for 4 weeks **a**. Tumors were harvested and weighed at 4 weeks post-injection **b**. **c**: qRT-PCR was conducted to detect the expression of MNX1-AS1, COMMD8, and miR-218-5p in tumor tissues. **d**: Immunohistochemistry was used to examine Ki-67 protein levels in tumor tissues. n = 5, error bars indicate SD. ^*^P < 0.05 *vs* shRNA-NC

The biological function of MNX1-AS1 in NSCLC progression was further investigated using in vivo tumorigenesis assays. Compared with the shRNA-NC group, the tumor volume and weight were significantly reduced in the sh-MNX1-AS1 group (*P* < 0.05, Fig. 5a, b). Moreover, the expression of MNX1-AS1, COMMD8 and Ki-67 were downregulated, while miR-218-5p expression was upregulated in xenograft tissues with MNX1-AS1 knockdown (*P* < 0.05, Fig. 5c, d). Collectively, these results confirmed that MNX1-AS1 knockdown inhibited tumorigenesis in vivo by modulating the miR-218-5p/COMMD8 axis.

## Discussion

Numerous studies have confirmed that lncRNAs play pivotal roles in NSCLC tumorigenesis, invasion, and metastasis. By regulating gene expression and modulating the activity of multiple signaling pathways, lncRNAs govern the malignant progression of NSCLC (Chen et al. 2016; Sun et al. 2016; Zang et al. 2016). Previous evidence has indicated that the lncRNA MNX1-AS1 is highly expressed in NSCLC. Silencing MNX1-AS1 dramatically impairs the proliferation, migration, and invasion abilities of NSCLC cells and induces cell apoptosis (Liu et al. 2019a). Mechanistically, MNX1-AS1 drives LUAD progression via the miR-34a/SIRT1 axis (Liu et al. 2022). In the present study, MNX1-AS1 was upregulated in NSCLC tissues, and high MNX1-AS1 expression correlated with poorer overall survival. Furtthermore, MNX1-AS1 functioned as an oncogene to positively regulate cell viability, migration, and invasion in NSCLC cells.

LncRNAs commonly exert functions by sponging miRNAs to modulate their expression. miR-218-5p has been reported to be downregulated in many human cancers (Li et al. 2024, 2021; Pan et al. 2020). In NSCLC, miR-218-5p is downregulated in tumor tissues and acts as a tumor suppressor by inhibiting cell proliferation and migration (Zhu et al. 2016). It has also been shown to restrain NSCLC cell growth by targeting TRIM9 (Wang et al., 2023b). In this study, miR-218-5p was identified as a direct downstream target of MNX1-AS1. MNX1-AS1 silencing significantly elevated miR-218-5p expression in H1299 and HCC827 cells. Clinically, miR-218-5p was significantly downregulated in NSCLC tissues compared with paired adjacent normal tissues, and a negative correlation was observed between miR-218-5p and MNX1-AS1.

Although COMMD8 has been reported to be upregulated in NSCLC and promote cell proliferation (Wang et al. 2020a), its upstream regulatory mechanism remains poorly elucidated. In the present study, COMMD8 was found to be negatively regulated by miR-218-5p. Rescue assays confirmed that COMMD8 overexpression partially reversed the tumor-suppressive effects caused by MNX1-AS1 knockdown. These findings provide new insights into the regulatory mechanism of COMMD8 and clarify the downstream signaling pathway of MNX1-AS1, suggesting that the oncogenic function of MNX1-AS1 is mediated via the miR-218-5p/COMMD8 axis.

Rescue experiments further revealed that the introduction of a miR-218-5p inhibitor or COMMD8 overexpression only partially reversed the tumor-suppressive effects induced by MNX1-AS1 knockdown, rather than restoring cell phenotypes to baseline levels. This phenomenon confers important biological implications. Firstly, MNX1-AS1 acts as a multifunctional oncogenic lncRNA, with a complex regulatory network characterized by ‘‘one-to-many’’ and ‘‘many-to-one’’ interactive patterns. Within the ceRNA regulatory cascade, beyond the validated miR-218-5p/COMMD8 axis, MNX1-AS1 sponges multiple functionally independent miRNAs, including those involved in the miR-34a/SIRT1 axis (Liu et al. 2022) and miR-527/BRF2 axis (Liu et al. 2019b). These parallel pathways coordinately drive malignant phenotypes of NSCLC cells. Rather than functioning as simple linear modules, these regulatory networks exert synergistic effects and provide compensatory backup mechanisms. Knockdown of MNX1-AS1 simultaneously abolishes the sponging effect on multiple downstream miRNAs, triggering broad-spectrum tumor-suppressive effects. In contrast, single inhibition of miR-218-5p or restoration of COMMD8 only rescues individual nodes within the whole regulatory network. Persistent activation of other parallel ceRNA axes consequently results in incomplete phenotypic reversal. Secondly, MNX1-AS1 may also facilitate oncogenic progression through ceRNA-independent mechanisms. For example, c-Myc transcriptionally activates MNX1-AS1, and MNX1-AS1 promotes the phase separation of IGF2BP1. This interaction strengthens the binding capacity of IGF2BP1 to c-Myc and E2F1 mRNAs, enhances mRNA stability, and ultimately accelerates cell cycle progression and proliferation (Zhu et al. 2022). MNX1-AS1 has also been shown to bind PABPC1 and activate the downstream Notch1 signaling pathway, thereby promoting NSCLC progression prog p (Liu et al. 2025). Such miRNA-independent regulatory modes cannot be rescued by miR-218-5p inhibition or COMMD8 overexpression, further explaining the incomplete phenotypic reversal observed in rescue assays. Thirdly, COMMD8 represents only one downstream effector of MNX1-AS1 and its biological function may be compensated by other homologous family members or alternative signaling pathways. Exogenous COMMD8 overexpression merely elevates the level of this single gene and cannot recapitulate the comprehensive regulatory function of MNX1-AS1 across the whole molecular network. Notably, compared with a previous study that initially identified the miR-218-5p/COMMD8 axis in hepatocellular carcinoma (Ji et al. 2019), the present study is the first to fully validate the complete regulatory axis in NSCLC. Multiple validations, including bidirectional rescue experiments, in vivo tumorigenesis assays, and clinical correlation analyses, further strengthen the reliability and tissue applicability of this regulatory mechanism in NSCLC.

Despite the novel findings of the present study, several limitations still exist. First, the single-center design and limited sample size may reduce the generalizability of the conclusions. Second, multivariate Cox regression was not performed due to incomplete clinical covariate data, which failed to validate the independent prognostic value of MNX1-AS1. Future studies will enlarge the sample size, collect complete clinical data and validate the findings in independent cohorts. Third, the lack of in vivo rescue experiments fails to confirm whether miR-218-5p inhibition or COMMD8 overexpression could reverse the tumor-suppressive effects induced by MNX1-AS1 knockdown in animal models. Subsequent studies will establish stably co-transfected cell lines for rigorous in vivo rescue validation. Fourth, the mechanistic exploration of the MNX1-AS1/miR-218-5p/COMMD8 axis remains insufficient. Only the canonical ceRNA regulatory mechanism was validated in this study, while alternative pathways or non-ceRNA modes by which MNX1-AS1 modulates COMMD8 expression were not explored. Furthermore, the specific downstream signaling pathways responsible for the biological functions of COMMD8 in NSCLC were not elucidated. The exclusive focus on this single ceRNA axis also omitted systematic screening of other potential MNX1-AS1-targeted miRNAs and their synergistic regulatory networks, which accounted for the partial phenotypic reversal observed in functional assays. To fill this mechanistic gap, follow-up studies will combine pathway inhibitor intervention and western blot analysis to identify COMMD8-associated downstream signaling pathways, and further verify the biological function of MNX1-AS1-centered regulatory network via multiple rescue assays.

In summary, MNX1-AS1 knockdown inhibits in vivo tumorigenesis of NSCLC via modulation of the miR-218-5p/COMMD8 axis, demonstrating that MNX1-AS1 exerts oncogenic effects on NSCLC progression through this molecular regulatory pathway.

## Data Availability

All data generated or analyzed during this study were included in the published article.
